# Transcriptional analyses provide new insight into the late-stage immune response of a diseased Caribbean coral

**DOI:** 10.1098/rsos.172062

**Published:** 2018-05-16

**Authors:** Lauren E. Fuess, Whitney T. Mann, Lea R. Jinks, Vanessa Brinkhuis, Laura D. Mydlarz

**Affiliations:** 1 Department of Biology, University of Texas Arlington, Arlington, TX, USA; 2 Florida Fish and Wildlife Conservation Commission, Fish and Wildlife Research Institute, 100 8th Avenue SE, St Petersburg, FL 33701, USA

**Keywords:** cnidarians, invertebrates, transcriptomics, ecoimmunology, invertebrate immunity, coral ecology

## Abstract

Increasing global temperatures due to climate change have resulted in respective increases in the severity and frequency of epizootics around the globe. Corals in particular have faced rapid declines due to disease outbreaks. Understanding immune responses and associated potential life-history trade-offs is therefore a priority. In the autumn of 2011, a novel disease of octocorals of the genus *Eunicea* was first documented in the Florida Keys. Termed Eunicea Black Disease (EBD), the disease is easily identified by the dark appearance of affected tissue, caused by a strong melanization response on the part of the host. In order to better understand the response of corals to EBD, we conducted full transcriptome analysis of 3 healthy and 3 diseased specimens of *Eunicea calyculata* collected from offshore southeast Florida. Differential expression and protein analyses revealed a strong, diverse immune response to EBD characterized by phagocytosis, adhesion and melanization on the part of the host. Furthermore, coexpression network analyses suggested this might come at the cost of reduced cell cycle progression and growth. This is in accordance with past histological studies of naturally infected hard corals, suggesting that potential trade-offs during infection may affect post-outbreak recovery of reef ecosystems by reducing both organismal growth and fecundity. Our findings highlight the importance of considering factors beyond mortality when estimating effects of disease outbreaks on ecosystems.

## 1. Introduction

Increasing global temperatures as well as other anthropogenic stressors have resulted in dramatic increases in the prevalence, frequency and severity of disease outbreaks [1,2]. Increases in disease have necessitated subsequent increases in our understanding of organismal immune systems and the physiological consequences of mounting an immune response [3–6]. These efforts have given rise to the field of ecological immunity, which is primarily concerned with understanding how immune responses vary between individuals and species, the dynamics of life-history–immune response trade-offs, and the consequences of immune response [7–11]. In particular, numerous studies focused on organismal level, reproductive consequences of immune response have been published in recent years [12–15]. Furthermore, many theories regarding the costs of immunity for individuals have also been developed [8,9,16–18]. Still many gaps exist in our knowledge of immunity and the consequences of immune trade-offs on community structures as a whole. Knowledge of the consequences of immune response on individuals and subsequently community structure is key to developing a robust understanding of how disease outbreaks shape community structure.

Marine ecosystems have arguably been the hardest hit by recent increases in epizootic outbreaks [1]. These rises in disease outbreaks in ocean ecosystems have resulted in significant die offs of urchins [19], corals [20] and shellfish [21]. Furthermore, many of these vulnerable invertebrate species are also essential to ecosystem function. Corals in particular form the structural and trophic basis of diverse coral reef ecosystems [22] and provide many important ecosystem services, such as serving as coastal protection and supporting fishing industries [23]. However, despite these grim predictions of increasing disease threat, and the importance of affected species, many gaps still exist in our knowledge of marine diseases. These include lack of information regarding the origins and spread of marine diseases, infectious stages and host ranges, and immune responses of host organisms [24].

Invertebrates possess a robust innate immune system consisting of three main processes: pathogen recognition, signalling pathways and effector responses [6]. Yet, while extensive research has revealed a framework for invertebrate immunity [17,25], many questions of host response, and potential trade-offs still remain. While it is clear that immunity comes at a cost, organisms cannot maintain optimal fecundity or longevity while also maintaining a robust immune system [16], few studies have investigated these trade-offs in vulnerable marine invertebrates. In part this is because the species most affected by disease outbreaks are non-model organisms such as corals [20] and echinoderms [26], for which there are limited existing resources [6,27]. Therefore, it is essential to increase efforts to understand immune function, and trade-offs between immunity and life history in these groups so as to better predict future ecosystem structures under rapid global change.

**Figure 1.**
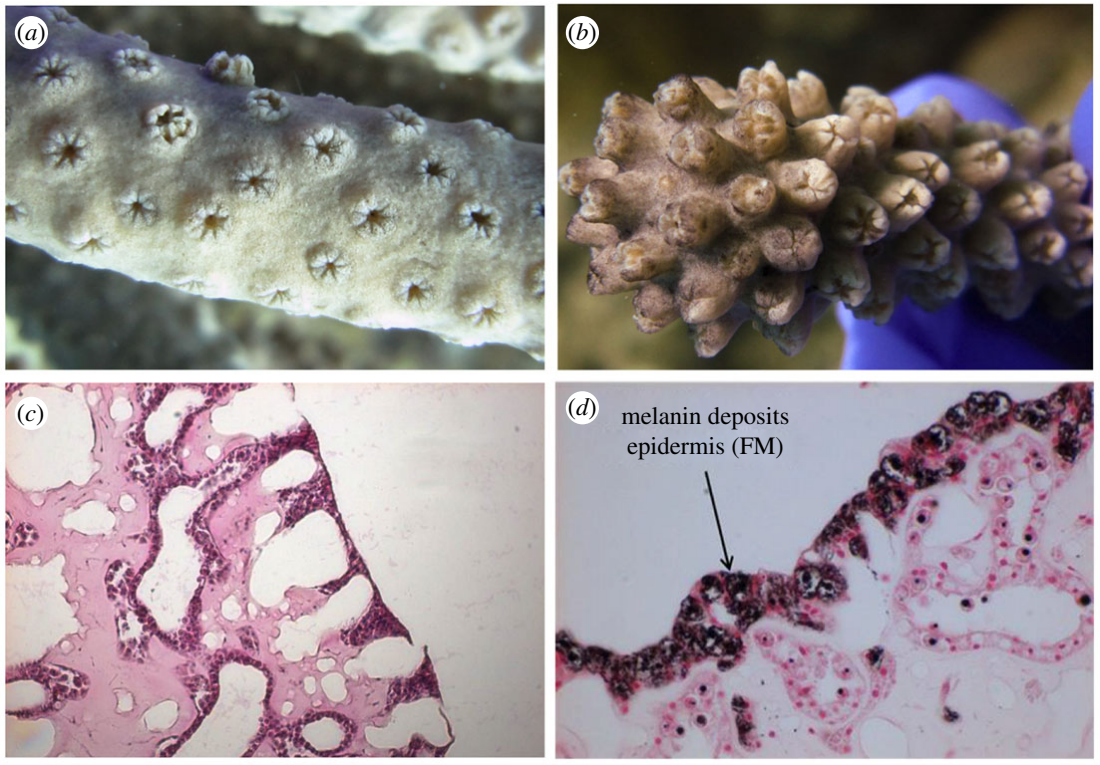
Macro and histological views of healthy (*a*,*c*) and diseased (*b*,*d*) *Eunicea*. (*a*) Close up of a healthy colony of *Eunicea* lacking any characteristic black pigmentation associated with the disease. (*b*) Close up of a colony of *Eunicea* infected with EBD; note the characteristic black pigmentation of the tissue. (*c*) Standard haematoxylin and eosin stained histology slides of apparently healthy sample showing good tissue organization and normal staining characteristics (pink staining) and (*d*) slides of tissue from an infected colony stained with Fontana-Masson showing the epidermis with melanin deposition.

In the autumn of 2011, a novel disease (Eunicea Black Disease; EBD) causing a heavily melanized appearance was observed affecting gorgonian corals of the genus *Eunicea* off the coast of Florida. To date EBD has been documented along the Florida Reef Tract from the Dry Tortugas National Park to offshore southeast Florida with prevalence ranging from 12 to 86% within the *Eunicea* community [28]. While the aetiological agent of this disease remains unknown, the disease is easily identifiable due to the darkening of affected tissue resulting from a strong melanization response (figure 1). Additionally, since its first observation, the disease has been reported in multiple other locations throughout the Caribbean (L.E.F. and L.D.M. 2017, personal observation). In order to document the response of these corals to this disease, and any potential trade-offs associated with the disease or immune-stimulated state, we conducted a transcriptional study of naturally affected *Eunicea calyculata* colonies. Here we document both the strong immune response of individuals to the disease that articulate the late stage or effector responses, and clear trade-offs with growth associated with the disease.

## 2. Methods

### 2.1. Sample collection

During October 2013, fragments of *Eunicea calyculata* were collected from 7 healthy and 7 diseased colonies from Southeast Coral Reef Evaluation and Monitoring Project site DC3, an octocoral reef, near Miami, FL (25° 50.526′ −80° 05.286′). Samples were collected while working with the Florida Fish and Wildlife Conservation Commission in compliance with Chapter 68B-8.016 of the Marine Special Activity License programme. Diseased corals were identified based on the presence of black tissue lesions and 3–5 cm fragments were cut from the affected areas. Fragments of the same size and approximate location were cut from normally pigmented colonies that did not show any signs of disease. Samples were immediately frozen in liquid nitrogen and shipped back to the University of Texas at Arlington on dry ice and stored at −80°C until processing for protein and transcriptomic analysis.

### 2.2. Protein analyses

Prior to protein processing, two small pieces (approximately 0.5 cm^2^) were removed from each sample and preserved for later RNA extraction. Proteins were extracted from all 14 samples by grinding the entire remaining sample in liquid nitrogen with a mortar and pestle. A small portion of this frozen powder was placed in a pre-weighed 1.5 ml centrifuge tube and reserved for melanin concentration analysis. Proteins were then extracted from the remaining powder on ice for approximately 45 min using approximately 2 ml of 100 mM sodium phosphate-buffered saline, pH 7.8. The remaining volume was centrifuged for 10 min at 4°C and 3500 r.p.m. in an Eppendorf centrifuge 5810R. The resulting coral extract was divided between 2 ml aliquots, flash frozen in liquid nitrogen, and stored at −80°C [29].

Prophenoloxidase (PPO) activity was estimated by diluting 20 µl of extract in 50 mM phosphate buffer, pH 7.0. Next, samples were incubated for 30 min in 25 µl of trypsin (0.1 mg ml^−1^). Just prior to the assay, 30 µl of 10 mM L-1,3-dihydroxphenylalanine (L-dopa, Sigma-Aldrich) was added to each sample. Absorbance was then read every 20 min at 490 nm. PPO activity was calculated as the change in absorbance per minute at the steepest part of the curve. A Red660 protein assay (G Biosciences, St Louis, MO) was used to determine total protein concentration in each sample. All colorimetric assays were run in duplicate on 96 well plates using a Synergy 2 multi-detection microplate reader and Gen5 software (Biotek Instruments, Winooski, VT, USA). The results of the PPO activity assay were then standardized by protein concentration as determined using a standard Red660 colorimetric assay [29]. Presented PPO activity results are representative of combined activity of proenzyme (PPO) activated with trypsin and existing active phenoloxidase within a sample.

In addition to PPO activity, total melanin concentration per sample was estimated using the portion of extract reserved for melanin analysis. The aliquot was lyophilized in a tarred 2 ml microcentrifuge tube overnight. At the end of this period, the total dry tissue weight in the aliquot was determined and a full spatula of glass beads (approx. 200 µl in volume) was added to each. Samples were vortexed for 10 s, 400 µl of 10 M NaOH was added to each tube, and the samples were vortexed for another 20 s. Tubes were then incubated for 48 h at room temperature in the dark and vortexed every 24 h. Following incubation, tubes were centrifuged at 1000 r.p.m. at room temperature for 10 min. 40 µl of supernatant was transferred to a ½ well UV plate (Costar, Corning Life Sciences, Lowell, MA, USA) and absorbance was recorded at 410 and 490 nm. Concentrations of melanin were estimated based on a standard curve of melanin dissolved in 10 M NaOH and are presented as mg of melanin per mg of tissue [29].

Assay results were analysed in SPSS software using standard ANOVAs with disease status as a factor. Protein data were tested for normality and outliers prior to statistical analysis. Data from both the PPO and melanin assays were normally distributed and required no transformation. Significance was determined as *p* < 0.05.

### 2.3. RNA extraction and sequencing

Total RNA was extracted from approximately 0.5 cm^2^ pieces of a subset of 3 fragments per group (healthy and diseased). Coral pieces were homogenized in Trizol (ThermoFisher) until all of the polyp tissue was removed from the gorgonin skeleton. Chloroform was added to the solution, incubated for 2–3 min and centrifuged at 12 000 × g at 4°C for 15 min. The aqueous phase was transferred to an RNeasy spin column and the extraction was completed per the RNeasy protocol (Qiagen). RNA quality was assessed using a 2100 BioAnalyzer (Agilent) and samples with RIN values greater than 8.0 were submitted for sequencing. Extracted RNA was submitted to the University of Texas Genome Sequencing Facility that created total RNA libraries using the NEBNext^®^ Ultra™ Directional RNA Library Prep Kit for Illumina^®^ (New England Biolabs, #E7420S) with poly-A tail selection. Libraries were constructed according to the manufacturer's protocol, with modifications to add custom barcodes during the ligation step. Samples were then sequenced at this facility on a single lane using an Illumina HiSeq 2500 platform with 100 base pair paired end reads.

### 2.4. Transcriptome assembly and differential expression analyses

Using the Trimmomatic software package [30] sequences were trimmed to remove adaptors and low quality reads. All sequences of 36 bp in length or less were discarded. Forward and reverse reads for all samples were concatenated together to create a reference forward and reverse read. Reference reads were fed into Trinity [31] for transcriptome assembly using default parameters. Non-host sequences were filtered out using methods described in [32]. Briefly, symbiont reads were removed by BLAT [33] comparison to a data file consisting of combined genomic and transcriptomic data from multiple species of *Symbiodinium*. The remaining transcriptome was filtered to obtain host only reads by BLAT comparison against a second meta-data file consisting of multiple cnidarian genomes (*O. favolata*, *Acropora digitifera* [34], *A. millepora* (http://genome.wustl.edu/genomes/detail/acropora-millepora/) and *Nematostella vectensis* [35]). Transcriptome completeness of the resulting host-only transcriptome was assessed via comparison to the publically available *Gorgonia ventalina* transcriptome [36]. A tblastx comparison of the two transcriptomes was conducted with a minimum *e*-value cut-off of 1 × 10^−5^.

Differentially expressed contigs were identified and normalized read counts generated using the Cufflinks software package with default parameters [37]. Log_2_fold change values for each contig were generated by comparing average normalized expression values between disease status groups. Significantly differentially expressed contigs were identified as those with an adjusted *p* < 0.05 between treatments.

### 2.5. Transcriptome annotation and gene ontology analyses

The full *E. calyculata* transcriptome generated from collected samples was annotated against the UniProtKB/Swiss-Prot and TrEMBL databases (blastx algorithm, 1.0 × 10^−5^ *e*-value threshold). Gene ontology (GO) was first assessed using the online PANTHER database [38] to determine relative abundance of GO terms. Additionally, to identify enriched functional groups that were characteristic of differentially expressed contigs, we conducted GO enrichment analysis using a Fisher's exact test with the R script GO MWU [39].

### 2.6. Coexpression network analysis

In order to identify groups of coexpressed contigs with potential correlation to disease status, the R package Weighted Gene Correlation Network Analyses [40] was used to analyse the full transcriptome. Normalized read counts for all contigs were log_2_ transformed. Using these values for all six samples, a single, signed network was built with manual network construction methods (parameters: soft power = 20, minimum module size = 180, deep split = 2, merged cut height = 0.25, minimum verbose = 3, cutHeight = 0.991). Module eigengene values (average expression of all contigs in a module) of each module were correlated to disease status, sample identity and protein analysis data using a bicor correlation. Modules with significant correlations (*p*-value ≤ 0.05) were selected for further analyses. GO enrichment analysis for each significant module was conducted using a Fisher's exact test with the R script GO MWU [39].

## 3. Results

### 3.1. Protein analyses

**Figure 2.**
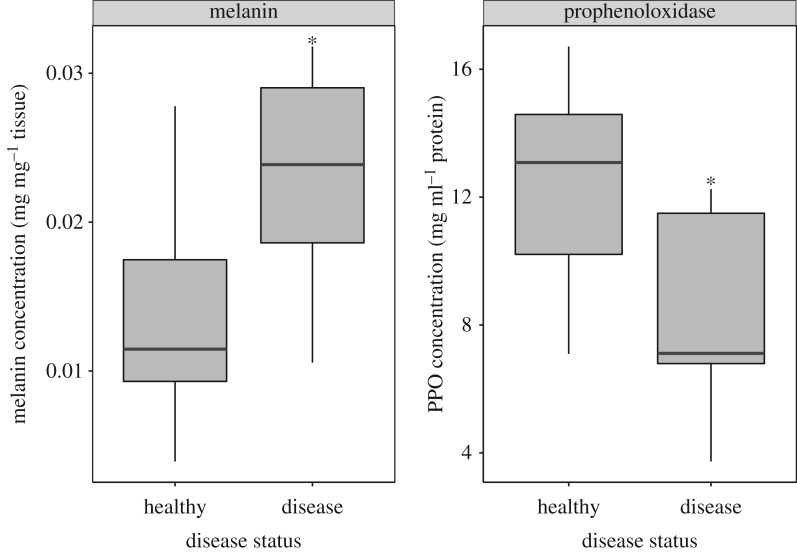
Results of biochemical assays measuring melanin concentration per mg tissue and prophenoloxidase protein activity (standardized by protein concentration). Infected samples had significantly higher melanin concentration (*p* = 0.031) and significantly lower prophenoloxidase activity (*p* = 0.040).

PPO activity was significantly reduced in diseased samples compared to those from healthy colonies (ANOVA, *p* = 0.040). In contrast, melanin concentration was significantly higher in diseased samples (ANOVA, *p* = 0.031, figure 2).

### 3.2. Transcriptome assembly

A total of 37 936 228 paired end reads were obtained from sequencing of all six samples (three healthy and three diseased). Full reads are available for download from NCBI (SRA PRJNA407366). Reads were assembled into a de novo transcriptome consisting of 95 665 contigs with an N50 value of 1364. Comparison of the full transcriptome to the UniProtKB/Swiss-Prot database resulted in annotation of 29 409 (approx. 31%) contigs.

Assessment of transcriptome completeness by comparison to the available *G. ventalina* transcriptome [36] suggested a robust transcriptome assembly. Blast results yielded 50 498 (52.8%) significant matches between the two transcriptomes. Furthermore, the two transcriptomes are relatively comparable in size, with the *G. ventalina* transcriptome consisting of 90 230 contigs, while our *E. calyculata* transcriptome was composed of 95 665 contigs. Finally both transcriptome assemblies had a similar N50 value, 1149 and 1364, for *G. ventalina* and *E. calyculata* respectively.

### 3.3. Differentially expressed contigs

**Figure 3.**
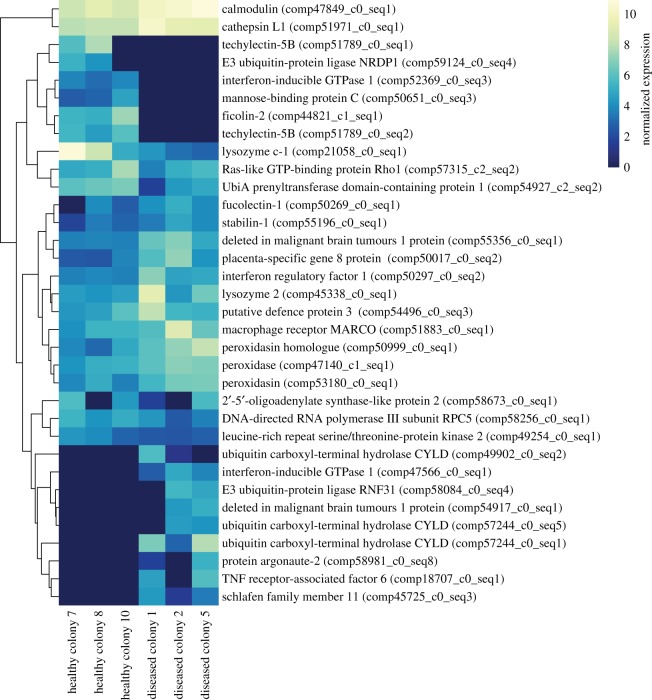
Heatmap of expression of all significantly differentially expressed immune contigs. Log2 transformed expression values are displayed for each sample. Colonies are ordered by treatment and contigs are clustered based on similarity in expression patterns. Contigs that were only expressed in one sample not shown.

Principal component analysis of patterns of expression among all six samples showed a clear division between healthy and diseased samples along the secondary principal component, and little division among the first principal component between samples. Disease status rather than colony identity appeared to be the primary factor contributing to sample variance in gene expression (electronic supplementary material, figure S1). Differential expression analyses comparing diseased to healthy samples found 1662 differentially expressed contigs: 1124 (approx. 68%) upregulated and 538 (approx. 32%) downregulated. 554 (approx. 33%) of the differentially expressed contigs were annotated (electronic supplementary material, file S1). A total of 37 contigs putatively involved in innate immune processes could be identified from this list (figure 3; electronic supplementary material, file S1). This included contigs involved in multiple different processes such as Toll-like receptor (TLR) signalling, complement cascade, antiviral responses, and the encapsulation and destruction of pathogens.

### 3.4. Gene ontology enrichment analyses

**Figure 4.**
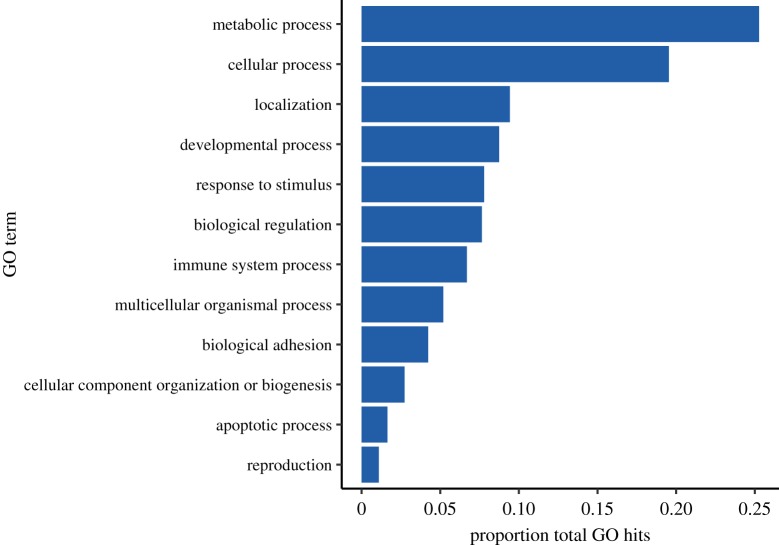
Top tier gene ontology breakdown of all annotated differentially expressed contigs. Shown as ratio of hits to total hits within a given category.

**Figure 5.**
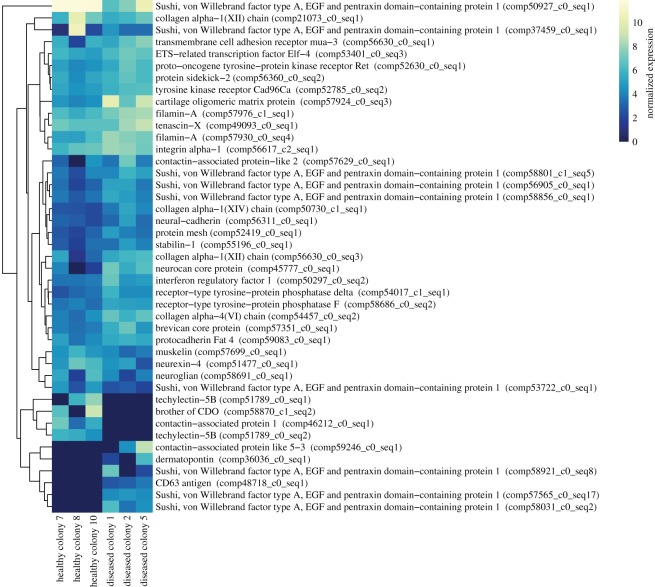
Heatmap of differentially expressed contigs that are involved in biological adhesion. Log2 transformed expression values are displayed for each sample. Colonies are ordered by treatment and contigs are clustered based on similarity in expression patterns. Contigs that were only expressed in one sample not shown.

Top tier GO breakdown of the 554 differentially expressed contigs revealed a large portion of contigs involved in response to stimulus, immune response and biological adhesion processes (figure 4). GO enrichment analyses of differentially expressed contigs found one significantly enriched term: biological adhesion. A total of 50 differentially expressed contigs were included in the biological adhesion GO term (figure 5). This included contigs with potential immune function such as those related to interferon responses, techylectins and integrins.

### 3.5. Coexpression analyses

**Figure 6.**
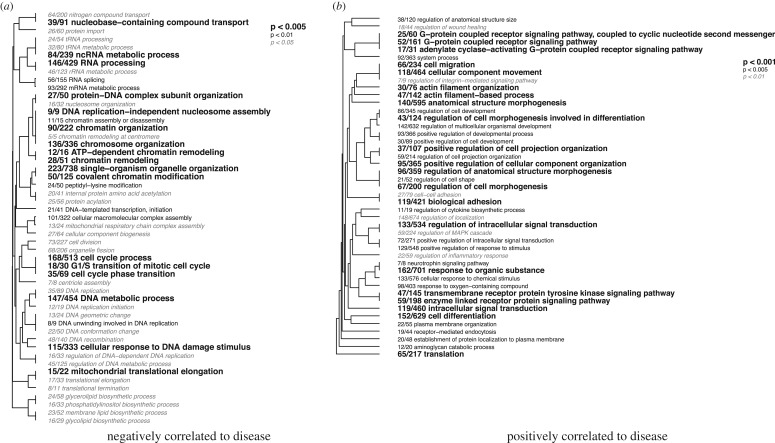
Hierarchical clustering of significantly enriched biological process gene ontology enrichment of the two modules correlated to disease status: (*a*) module 4 (negatively correlated) and (*b*) module 19 (positively correlated).

Coexpression analyses of all expressed contigs using WGCNA resulted in a network composed of 29 modules ranging in size from 190 to 3252 contigs each. Of these 29 modules, 2 were significantly correlated to disease status, modules 4 (*r* = −0.93, *p* = 0.008) and 19 (*r* = 0.98, *p* < 0.001) (electronic supplementary material, figure S2). These two modules were significantly enriched for multiple biological process GO terms, 50 terms for module 4 and 114 terms for module 19 (*p* < 0.05; figure 6, electronic supplementary material, file S3). Module 4, which was negatively correlated to disease status, was significantly enriched with terms involved in RNA and DNA processing, as well as cell cycle progression (electronic supplementary material, figure S3). Module 19, which was positively correlated to disease status, was significantly enriched with immune terms such as inflammatory response, regulation of MAPK cascade, receptor mediated endocytosis and regulation of wound healing. Additionally this module was significantly enriched for terms involved in cell signalling such as G-protein coupled receptor signalling and regulation of intracellular signalling, as well as terms involved in cell adhesion such as cell–cell adhesion and biological adhesion (electronic supplementary material, figure S4).

An additional four modules were correlated with either melanin concentration or PPO activity (electronic supplementary material, figure S2). Module 9 was significantly negatively correlated to melanin activity (*p* *=* 0.03, *r* = −0.83). This module was not significantly enriched for any biological process GO terms. Three modules were significantly correlated to PPO activity: module 12 (*p* = 0.03, *r* = 0.86), module 14 (*p* = 0.02, *r* = 0.89) and module 21 (*p* = 0.04, *r* = −0.84). Module 14 was also significantly correlated to healthy colony 8. Again, none of these modules were significantly enriched for any biological process GO terms.

## 4. Discussion

Coastal ecosystems such as coral reefs have experienced rapid declines due to increasing global temperatures and other anthropogenic stressors [1,2]. This outbreak of yet another novel coral disease is further evidence of rapidly declining ocean conditions and coral health. This disease in particular is clearly characterized by darkly pigmented tissue resulting from a strong immune response dominated by melanin deposition (figure 1). The disease phenotype is in fact the result of a melanization response as evidenced by the results of our assays (figure 2). Furthermore, patterns of gene expression among samples were predominately driven by disease status rather than colony identity (electronic supplementary material, figure S1). Differential expression and GO analyses reveal significant changes in numerous immune and adhesion contigs underlying these responses. Finally, coexpression analyses suggest that this response may come at the cost of reduced cell division and growth. Here we further examine the immune response and associated fitness trade-offs of the host *E. calyculata* using transcriptional analyses.

### 4.1. Infected *E. calyculata* mount a strong immune response characterized by melanization to an unknown pathogen

Black pigmented diseased tissue *E. calyculata* demonstrated a clear immune response as evidenced by both protein and gene expression data. Biochemical analysis of protein samples revealed that infected tissue had significantly lower PPO activity than apparently healthy tissue. Consequently, infected tissue also had higher melanin concentration. This is as would be expected for a late-stage immune response. Melanization is the end product of the PPO cascade in invertebrates [41,42] and has been previously documented as an important component of the cnidarian immune response [43,44]. Therefore, the observed response is likely indicative of previous rapid melanization and subsequent reduction in PPO availability or activity. These findings here add to existing ecological observations and histology results that suggest that melanization is a key component of the response of *E. calyculata* to EBD.

Supporting our protein results, we found contigs representative of various components of innate immunity to be differentially expressed, including components of the TLR signalling pathway, complement cascade, antiviral response, phagocytosis and antioxidant activity. TLR signalling is an important mechanism by which hosts detect pathogens and activate immune responses [45]. Here we document 8 differentially expressed contigs involved in or downstream of this pathway, including two copies of the signalling protein deleted in malignant brain tumour protein 1 (DMBT1) which is regulated by TLR signalling and has roles in pathogen recognition [46]. This protein has previously been shown to be an important part of the coral immune response in multiple studies and has been linked to host production of antimicrobial compounds [47,48]. Other TLR-related contigs we observed to be differentially expressed included tumour necrosis factor receptor-associated factor 6 [49,50], and contigs involved in the negative regulation of TLR signalling, E3 ubiquitin-protein ligase RNF31 [51] and 4 copies of ubiquitin carboxyl-terminal hydrolase CYLD [52]. Additionally, we observed upregulation of the protein argonaute 2, an antiviral protein that has previously been described as part of the response of acroporid corals to white diseases [53].

In addition to differential expression of conventional immune contigs, the biological process GO term *biological adhesion* was significantly enriched in our dataset. The enrichment of biological adhesion as a response to a pathogen is not surprising, as cellular adhesion is a basic component of multiple innate and adaptive immune responses [54–57]. Specifically, cellular adhesion is an important characteristic of the PPO activating system that results in the formation of melanin [25,56] that can be used to encapsulate pathogens for later phagocytosis [58]. Biological adhesion is also often associated with phagocytic response [25,55,56]. Interestingly, the majority of immune-related differentially expressed contigs were in fact related to phagocytosis and antioxidant responses. This included several contigs involved in recognition and agglutination of pathogens for later phagocytosis such as MARCO, techylectin-5B, stabilin-1, fucolectin-1 and DMBT1 [46,59–62], as well as lysozyme and peroxidase contigs which can be associated with phagocytosis [63]. Upregulation of antioxidants and lysozymes, which are frequently associated with degradation of phagocytized particles, is further suggestion of ongoing phagocytic processes [64–67]. Together these patterns of expression strongly support our hypothesis that diseased samples are undergoing a late-stage immune response characterized by phagocytosis of melanized particles and pathogens. Therefore, despite our limited sample size, our combination of protein data and robust transcriptomic analyses provides a clear and unified picture of late-stage immune responses in cnidarians.

Finally, it should be noted that the transcriptomic responses documented here share a great deal of overlap with a number of other transcriptomic studies of cnidarian immune responses [47,48,53,68,69]. Many of these studies document changes in a core subset of contigs that are shared across cnidarian species in response to both pathogenic challenge and immune stimulation. This suggests a common, conserved role of a robust innate immune response characterized by pathways such as the TLR signalling pathway in the cnidarian immune response.

### 4.2. *Eunicea calyculata* undergo a fitness trade-off during infection

GO enrichment analyses of the two modules that were significantly correlated to disease status demonstrated a trade-off associated with immune response. The module positively correlated to disease status was enriched with immune system and signalling processes, while the one negatively correlated to disease status was enriched with processes associated with cell cycle progression, including DNA and RNA processes. These findings were corroborated by examination of patterns of expression of contigs included in these two modules (figure 6 and electronic supplementary material, figures S3 and S4), suggesting that cellular division and growth may be impacted during a pathogenic infection of *E. calyculata*. Decreases in cell cycle and other like processes have the potential to detrimentally impact growth of the organism as a whole. As colonial organisms, octocorals such as *E. calyculata* undergo modular, iterative growth via replication of polyps through asexual budding [70,71]. Furthermore, asexual reproduction is dependent on proliferative cells; therefore decreases in cellular growth may have impacts on the growth, size, and ultimately fitness of the whole organism [72]. However the effects of decreased cell cycle progression may not only result in decreased size of the organism, but also potentially organism fecundity. Growth and size are an important determinant of fecundity, and therefore fitness, in colonial organisms such as gorgonian corals. Many gorgonian corals must maintain a certain colony size to reproduce [73,74], and larger colonies have larger reproductive outputs [74]. Subsequently, in theory, reduction in growth during disease would correspond to reduced long-term fecundity and fitness. While this is the first documentation of transcriptomic evidence of the potential consequences of disease on reproductive output in a cnidarian, it is in accordance with past observations reporting reduced fecundity (measured as amount of gravid polyps) in a hard coral infected with disease [75]. Therefore, our findings here provide new molecular context to previously published findings that disease comes at the cost of fecundity in corals.

It should be noted that samples used for transcriptomic analysis of diseased colonies were collected from heavily melanized lesion locations. This leaves the potential that observed signals are the result of either death response or the cellular stress response. However, we observed little differential expression of apoptotic, death or autophagic pathways in our analysis. Similarly we saw little evidence of the cellular stress response, particularly in comparison to other similar studies that document this as part of the cnidarian disease response [69]. Additionally these lesions often affect the majority of diseased colonies. Therefore, we are confident that the trends discussed here are relevant and have important potential ecological consequences.

Our findings documented here point to an ecologically relevant potential trade-off. Increasing sea surface temperatures as a result of climate change have resulted in increases in the prevalence of coral disease [76]. During active infection, our findings suggest that *E. calyculata* may reduce cell cycle progression, growth and fecundity. Therefore, as disease outbreaks become more common and individuals are more frequently infected, *E. calyculata* growth, and fecundity, may be impacted. This could have implications beyond individual corals as self-seeding is one of the most important sources of recruitment on a recovering reef [73,77]. Regardless, as *Eunicea* corals are relatively slow growing under normal conditions [78,79], recovery times will be slow, and even slower still should our findings here regarding reduced expression of growth related processes following disease be corroborated by further investigation. Therefore, mortality of *Eunicea* and reduced growth of remaining colonies may lead to displacement of the species by faster growing species such as those from the genus *Antillogorgia* [80].

## 5. Conclusion

The results presented here are some of the first transcriptomic evidence to corroborate past field and histological observations of the growth and fecundity consequences of disease in cnidarians. Furthermore, our documentation of the response of *E. calyculata* to disease joins a growing body of literature reporting shared transcriptomic responses of cnidarians to pathogenic infection and immune stimulation. The severity of this disease, robustness of the associated immune response, and potential related fitness trade-offs cast a grim outlook for the future of *Eunicea* octocorals on Caribbean reefs. As climate change effects increase and with it the frequency of disease outbreaks affecting numerous species [81], resilience of these communities may decrease, resulting in decreases or shifts in octocoral communities on reefs [82]. Therefore, it is essential to continue to explore our findings regarding the consequences of disease on growth and fecundity of individual corals. Increasing understanding of these potential trade-offs will improve our ability to understand species and community level effects of disease beyond mortality. Our findings open the door to future research that will increase our ability to predict coral community trajectories under increasing pressure from disease and climate change.

## Supplementary Material

Supplementary File 1

## Supplementary Material

Supplementary File 2

## Supplementary Material

Supplementary File 3

## Supplementary Material

Supplementary Figure 1

## Supplementary Material

Supplementary Figure 2

## Supplementary Material

Supplementary Figure 3

## Supplementary Material

Supplementary Figure 4

## Data accessibility

Raw RNAseq reads are available on NCBI GenBank (SRA PRJNA407366).
