# Supplementary figures and images for: Transcriptional analyses provide new insight into the late-stage immune response of a diseased Caribbean coral
Source: R Soc Open Sci. 2018 May 16;5(5):172062. doi: 10.1098/rsos.172062 (PMC5990752; doi:10.1098/rsos.172062)

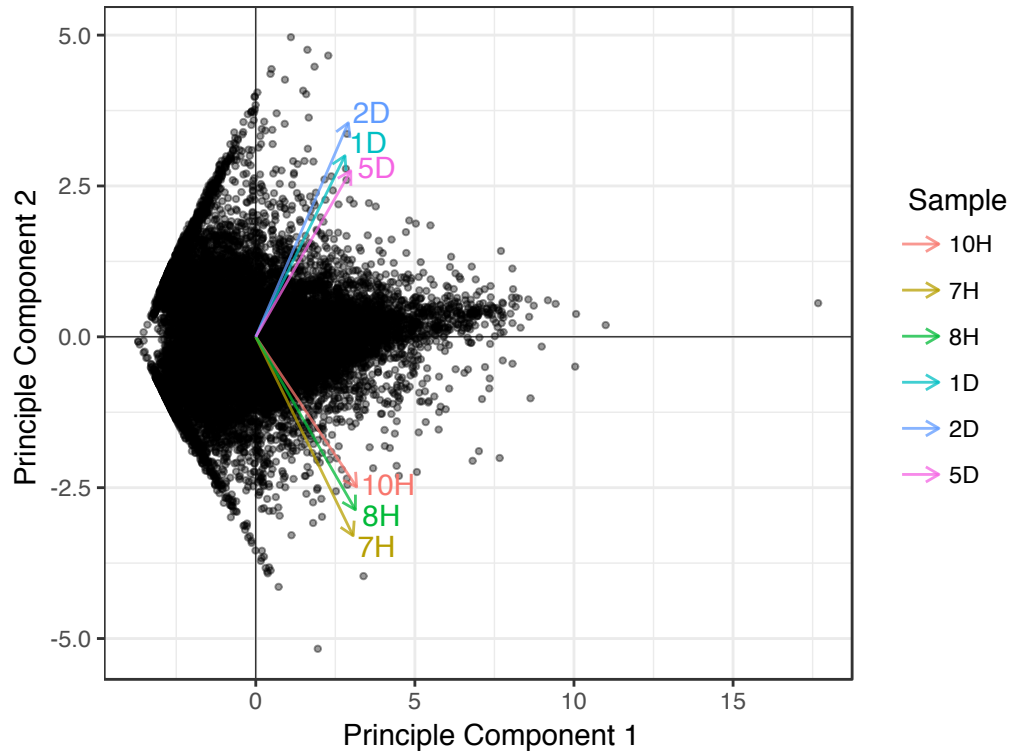

Supplement: Supplementary Figure 1 [file rsos172062supp4.pdf]

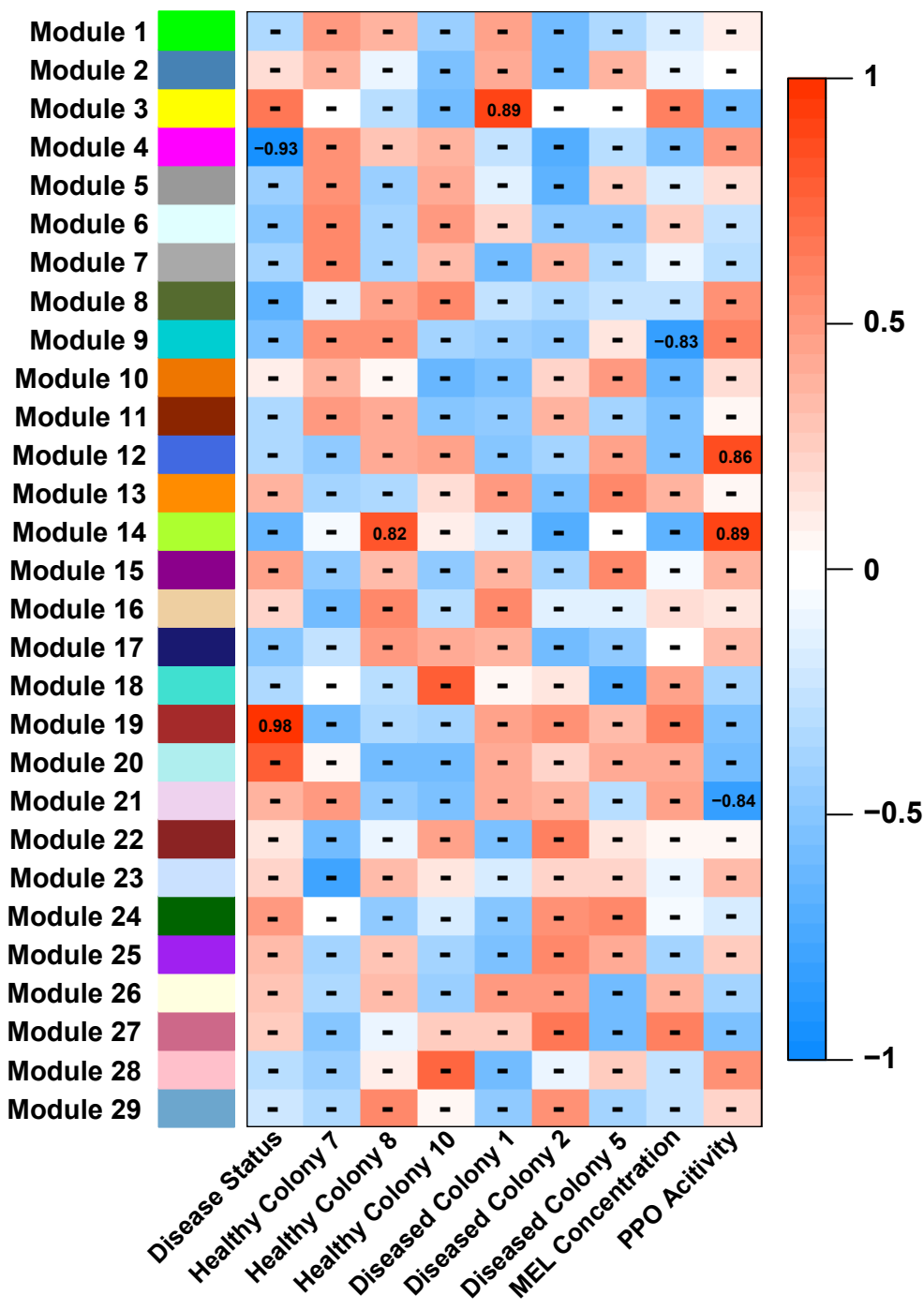

Supplement: Supplementary Figure 2 [file rsos172062supp5.pdf]
