# Supplementary material for: Transcriptional analyses provide new insight into the late-stage immune response of a diseased Caribbean coral
Source: R Soc Open Sci. 2018 May 16;5(5):172062. doi: 10.1098/rsos.172062 (PMC5990752; doi:10.1098/rsos.172062)
Supplement: Supplementary Figure 3 [file rsos172062supp6.pdf]

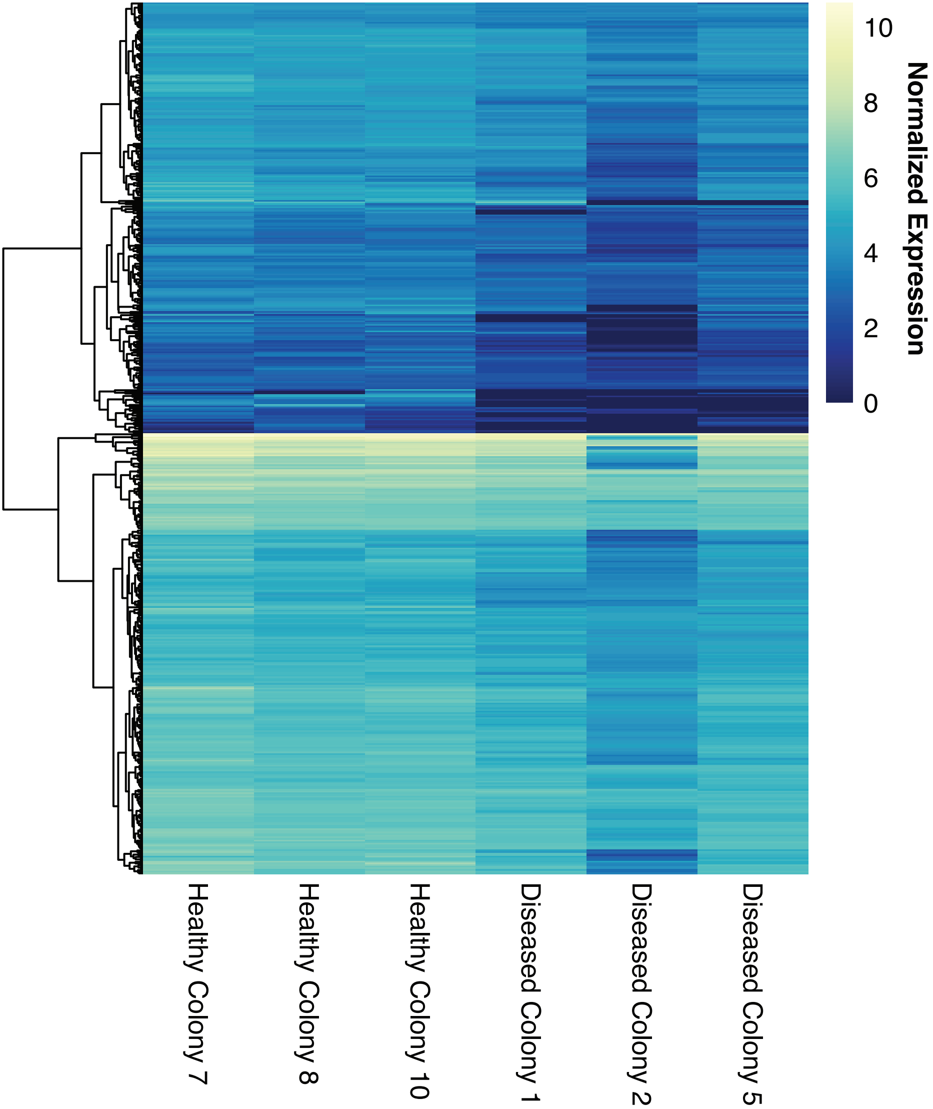

Normalized Expression
10
8
6
4
2
0
Healthy Colony 7
Healthy Colony 8
Healthy Colony 10
Diseased Colony 1
Diseased Colony 2
Diseased Colony 5
