# Supplementary material for: Transcriptional analyses provide new insight into the late-stage immune response of a diseased Caribbean coral
Source: R Soc Open Sci. 2018 May 16;5(5):172062. doi: 10.1098/rsos.172062 (PMC5990752; doi:10.1098/rsos.172062)
Supplement: Supplementary Figure 4 [file rsos172062supp7.pdf]

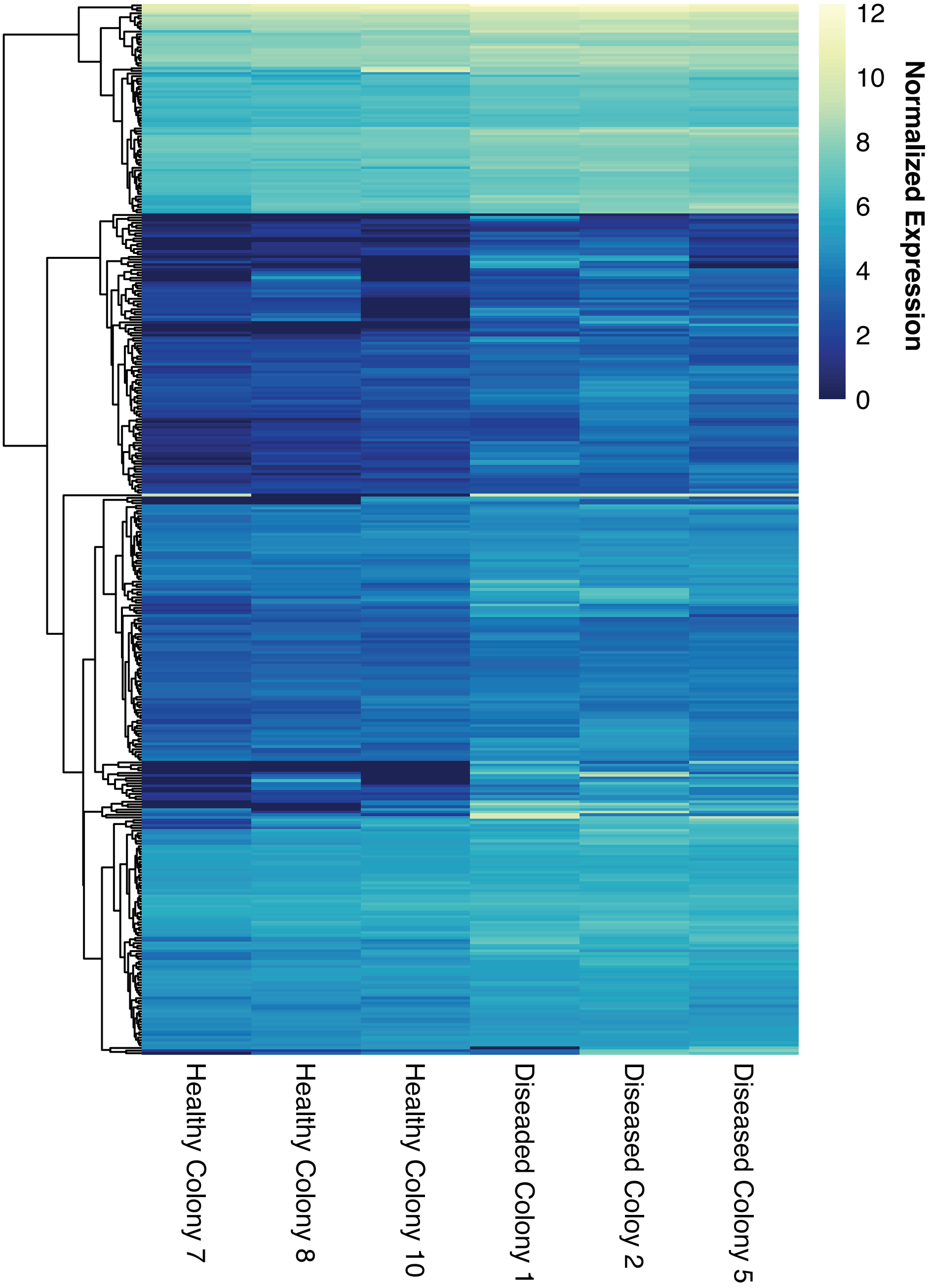

12
10
8
6
4
2
0
Normalized Expression
Healthy Colony 7
Healthy Colony 8
Healthy Colony 10
Diseaded Colony 1
Diseased Coloy 2
Diseased Colony 5
